# *Spag6* Mutant Mice Have Defects in Development and Function of Spiral Ganglion Neurons, Apoptosis, and Higher Sensitivity to Paclitaxel

**DOI:** 10.1038/s41598-017-08739-8

**Published:** 2017-08-17

**Authors:** Xiaofei Li, Lei Xu, Gaoying Sun, Xianmin Wu, Xiaohui Bai, Jianfeng Li, Jerome F. Strauss, Zhibing Zhang, Haibo Wang

**Affiliations:** 1 Otolaryngology-Head and Neck Surgery, Provincial Hospital Affiliated to Shandong University, Jinan, PR China; 2 Shandong Provincial Key Laboratory of Otology, Jinan, PR China; 3 Department of Obstetrics and Gynecology, Virginia Commonwealth University, Richmond, VA 23298 USA; 4 Department of Biochemistry and Molecular Biology, Virginia Commonwealth University, Richmond, VA 23298 USA; 5 Department of Otolaryngology-Head and Neck Surgery, First Affiliated Hospital of Wenzhou Medical University, Wenzhou, PR China

## Abstract

Mammalian Sperm Associated Antigen 6 (SPAG6) is the orthologue of *Chlamydomonas PF16*, a protein localized in the axoneme central apparatus. Recent studies showed that *Spag6* has a role in brain neuronal proliferation and differentiation. The mammalian spiral ganglion neurons (SGNs) are specialzed bipolar neurons in the inner ear. However, the role of SPAG6 in SGN has not been elucidated. Therefore, We hypothesized that a *Spag6* knockout would affect the development and function of SGNs. We utilized *Spag6*-deficient mice and SGN explants to define the role of SPAG6. On postnatal day 30 (P30) mutant mice had lower SGN density compared to their wild-type littermates, and more apoptosis was evident in the mutants. Increased Bax expression, a disturbed distribution of cytochrome c, and cleaved caspase-3 positive staining indicated that increased apoptosis involved a mitochondrial pathway. Transmission electron microscopy revealed abnormalities in the ultrastructure of mutant SGNs as early as P7. *In vitro*, lack of SPAG6 affected the growth of neurites and growth cones. Additionally, SPAG6 deficiency decreased synapse density in SGN explants. Finally, *Spag6* mutant SGNs were more sensitive to the microtubule stabilizing agent, paclitaxel. These findings suggest that *Spag6* plays a crucial role in SGN development and function.

## Introduction

The mammalian spiral ganglion neurons (SGNs) are specialized bipolar neurons that transmit auditory information from hair cells (HCs) to the central nervous system. SGNs originate from the cochleovestibular ganglion and then mature within the prosensory domain of the cochlea^1^. Finally, the SGNs extend peripheral dendrites and make synaptic contact with the base of hair cells, and their axons are bundled together to form the auditory portion of eighth cranial nerve connecting with brain^2^. These bipolar neurons are the first neurons in the auditory system to fire an action potential, and supply all of the brain’s auditory input. Therefore, SGNs are essential for auditory function.

Mammalian Sperm Associated Antigen 6 (SPAG6) is the orthologue of *Chlamydomonas PF16*, which encodes a protein localized in the axoneme central apparatus, and regulates flagella/cilia motility. Besides being expressed in cilia-bearing tissues like lung and brain, Hamada *et al*. reported that *Spag6* is highly expressed in the embryonic chick spinal cord^3^, and Zhao *et al*. found a role for *Spag6* in regulating neuronal migration during mouse brain development^4^ and in the process of neuronal proliferation and differentiation^5^. These studies imply a potentially important role for SPAG6 in neuron development. However, it is not known if *Spag6* has a role in SGNs, the specialized bipolar neurons in the inner ears.

Other studies demonstrated that *SPAG6* has a role in some malignant tumors such as non-small cell lung cancers^6^ and acute myelogenous leukemia^7^. A striking upregulation of *Spag6* as well as the three other genes was observed in CALM/AF10-positive leukemias^8^. Down-regulation of *SPAG6* gene expression was correlated with a better prognosis and survival^7^. *SPAG6* silencing inhibited the growth of malignant myeloid cell lines, SKM-1 and K562, consistent with a role for *SPAG6* in malignant myeloid cell proliferation and apoptosis^9^.

Apoptosis is a major type of regulated cell death^10^. The protease activity of caspases is essential for execution of apoptosis^11^. In broad terms, caspases can be activated through one of two pathways - the extrinsic (also called death receptor) pathway, and the intrinsic (also called mitochondrial) pathway. The intrinsic (or mitochondrial) pathway of apoptosis requires mitochondrial outer membrane permeabilization (MOMP). BAX activity triggers MOMP while the anti-apoptotic Bcl-2 family proteins counteract this pathway. Therefore, The balance between the Bax and Bcl-2 is very important. Following MOMP, the mitochondrial intermembrane space protein, cytochrome c, is released into the cytosol, which activates the cascade of caspases leading to cell apoptosis.

Paclitaxel is a cancer chemotherapy drug that targets microtubules. It binds to β-tubulin subunits and stabilizes the microtubule polymers. Chromosomes are thus unable to achieve a metaphase spindle configuration in the presence of paclitaxel. This blocks the progression of mitosis, and the prolonged activation of the mitotic checkpoint triggers apoptosis^12, 13^. It has been reported that human SPAG6 expression is increased in several human malignancies^14–16^. It has also been speculated that overexpression of SPAG6 might be the reason why some cancers are resistant to microtubule-targeting drugs, such as paclitaxel. Paclitaxel also produces ototoxicity in rat cochlear organotypic cultures. Our previous study also showed an increased cell death rate of the *Spag6*-deficient mouse embryonic fibroblasts in response to paclitaxel^17^.

To further explore the role of SPAG6 in SGNs, We took advantage of *Spag6* -deficient mice, SGN explants and organ culture from *Spag6*-deficient mice and their wild-type littermates. Surprisingly, SGNs in mutant mice presented apoptosis as early as postnatal 30 days (P30). Although we did not observe apoptosis of SGNs on P14, transmission electron microscopy revealed abnormalities in the ultrastructure of mutant SGNs at P7. SGN explant cultures showed that lack of SPAG6 affects neurite number, length and average areas of SGNs in explants. SPAG6 deficiency also decreased synapse density in SGN explants and impaired the growth of growth cones. Finally, *Spag6* mutant SGNs were more sensitive to paclitaxel. These findings suggest that *Spag6* plays a crucial role in SGN development and function.

## Materials and Methods

### Animals

All experiments were approved by the Animal Care Committee of Shandong University, China, on the care and use of Laboratory Animal for Research Purposes. All methods used in the study were performed in accordance with the guidelines and regulations of the U.S. National Institutes of Health (NIH) Office of Laboratory Animal Welfare (OLAW) and the Public Health Service (PHS) Policy on the Humane Care and Use of Laboratory Animals. *Spag6* mutant mice were obtained as described in a previous study^18^.

### Tissue preparation for frozen sections

Cochlea were isolated from ears of control (n = 4) and *Spag6* mutant mice (n = 4). After dissection, cochlea were fixed in 4% PFA overnight and then decalcified in 10% EDTA for 3 days and dehydrated with different concentrations of sucrose. After dehydration, tissues were embedded in O.C.T. (Sakura) for frozen sectioning and immunofluorescence staining.

### SGN organotypic cultures

Postnatal 3-day-old (P3) *Spag6* mutant and wild-type mice were used for organotypic culture. The dissection procedures and the three-dimensional (3D) culture media were carried out as described in a previous study^19^.

### Cochlear organotypic cultures and paclitaxel treatment *in vitro*

Cochlear organotypic cultures were prepared from P3 *Spag6* mutant and wild-type mice following procedures similar to those described previously^20^. Briefly, the cochlea was removed and the entire organ of Corti and SGN transferred on to sterile Hank’s Balanced Salt Solution (Hyclone, USA) on a flat ice pack. The upper basal turn was cut and adhered onto a 10 mm glass coverslip precoated with CellTak (BD Biosciences, USA) and cultured in 48-well plate (Greiner Bio-One, Germany) in full medium (FM) as described in previous study^19^. The cultures were placed in an incubator at 37 °C in 5% CO_2_ overnight. On the following day, fresh FM was added alone or in the presence of various concentrations of paclitaxel. The paclitaxel stock solution was at a concentration of 7 mM and diluted in FM to a final paclitaxel concentration of 1 μM, 10 μM or 30 μM. Cochlear explants were cultured in 5% CO_2_ at 37 °C in a humidified atmosphere for 48 hours. Then the cultures were fixed by 4% paraformaldehyde (PFA) for following use.

### Immunostaining

The procedures were performed as described in a previous study^21^. The samples(frozen sections and cultured tissues) were incubated with different primary antibodies: SPAG6^21^, cleaved caspase-3 (1:400, Cell Signalling, USA, cytochrome C (1:400, Cell Signalling, USA) β-tubulin Ш (1: 1000, Neuromics, USA, a neuron-specific marker), neurofilament (1: 1000, Millipore, USA, a neuron-specific marker) and synaptophysin (1: 1000, Millipore) overnight. The next day, tissues were incubated with FITC conjugated or TRITC-conjugated (1: 1000, Invitrogen) secondary antibody along with DAPI (1: 800, Sigma-Aldrich) or phalloidin (1: 1000, Sigma Aldrich) for 1 hour. Then, the coverslips were mounted and observed under a laser scanning confocal microscope (Leica, Germany).

### Reverse transcription-polymerase chain reaction (RT-PCR)

The cochlear modiolus were collected from postnatal 1 day mutant and wild-type mice and total RNA was isolated from each tissue sample using Trizol reagent according to the manufacturer’s instructions (Invitrogen, Carlsbad, CA, USA). The cDNA was synthesized from each RNA sample using cDNA Synthesis Kit (Ta Kara Bio, Siga, Japan). Primers used for RT- PCR are reported previously^21^.

### Transmission electron microscopy

Briefly, animals were decapitated under deep anesthesia, and the cochlear tissue was removed, washed quickly with PBS, and immediately placed in 3% glutaraldehyde fixative solution (pH 7.4). The tissue was trimmed according to conventional TEM sample preparation methods, followed by rinsing, and fixation in 1% osmic acid (OsO4), rinsing, dehydration, and embedding in Epon812. Semi- and ultra-thin radial sections were cut from the upper middle turns (as illustrated in Fig. 2A) with lead citrate and uranyl acetate staining. The sections were observed using a transmission electron microscope (JEOL-1200EX) in JiNan WeiYa Bio-Technology Co, Ltd. (Jinan, China).

### Western Blotting

The cochlear modiolus were collected quickly and placed on ice. The modiolus proteins were extracted for Bax and Bcl-2 detection. Briefly, the protein was extracted from cochleae using lysis buffer and protein was measured using the BCA protein assay kit. 30 μg of each protein sample was separated in 12% SDS-PAGE gels. The primary antibodies were anti-Bax (1:1000, Cell Signalling, USA) and anti-Bcl-2 (1:1000, Cell Signalling, USA. The relative optical density ratio of Bcl-2 and Bax to actin were calculated with Image J software.

### Image Analysis

Confocal images of SGNs were taken using a Leica SPE confocal fluorescence microscope (Leica). Z-stack images were taken at 1 μm intervals to span the samples. The number, length and area of the neurite outgrowth were evaluated separately as described in previous studies^19^ with slight modifications. In order to reduce the error caused by different size of SGN bulk, the number and area of SGN neuritis were normalized by dividing each SGN bulk size (μm^2^). The data were calculated by Image J software. As for cochlear organotypic cultures, the fascicles of auditory nerve fiber (ANF) bundles projecting out from the SGN to the organ of Corti were counted across the width (175 μm) of the field under 63x oil lens. All the fibers were counted in the same region in the middle of the cochlear culture. For calculation of SGN density, only the upper middle turn in the frozen section through the modiolus was analyzed. β-tubulin Ш, a neuron-specific marker, was used for SGN staining. The SGNs were counted and SGN density was calculated as the ratio of SGN number to the triangular area where SGN localized.

### Statistical analysis

At least four individual experiments were conducted for each experimental condition. Western blotting was repeated on three biological replicates for each experimental condition. Data are presented as means ± standard deviation. Comparisons between two groups were tested by Student’s t-test. The rate comparison between two groups was analyzed by chi-square test. Pairwise comparison between three or multiple groups were analyzed using a one-way ANOVA followed by Newman–Keuls post hoc analyses (GraphPad Prism 5 software). P < 0.05 was considered statistically significant.

## Results

### SPAG6 is expressed in mouse SGNs

**Figure 1:**
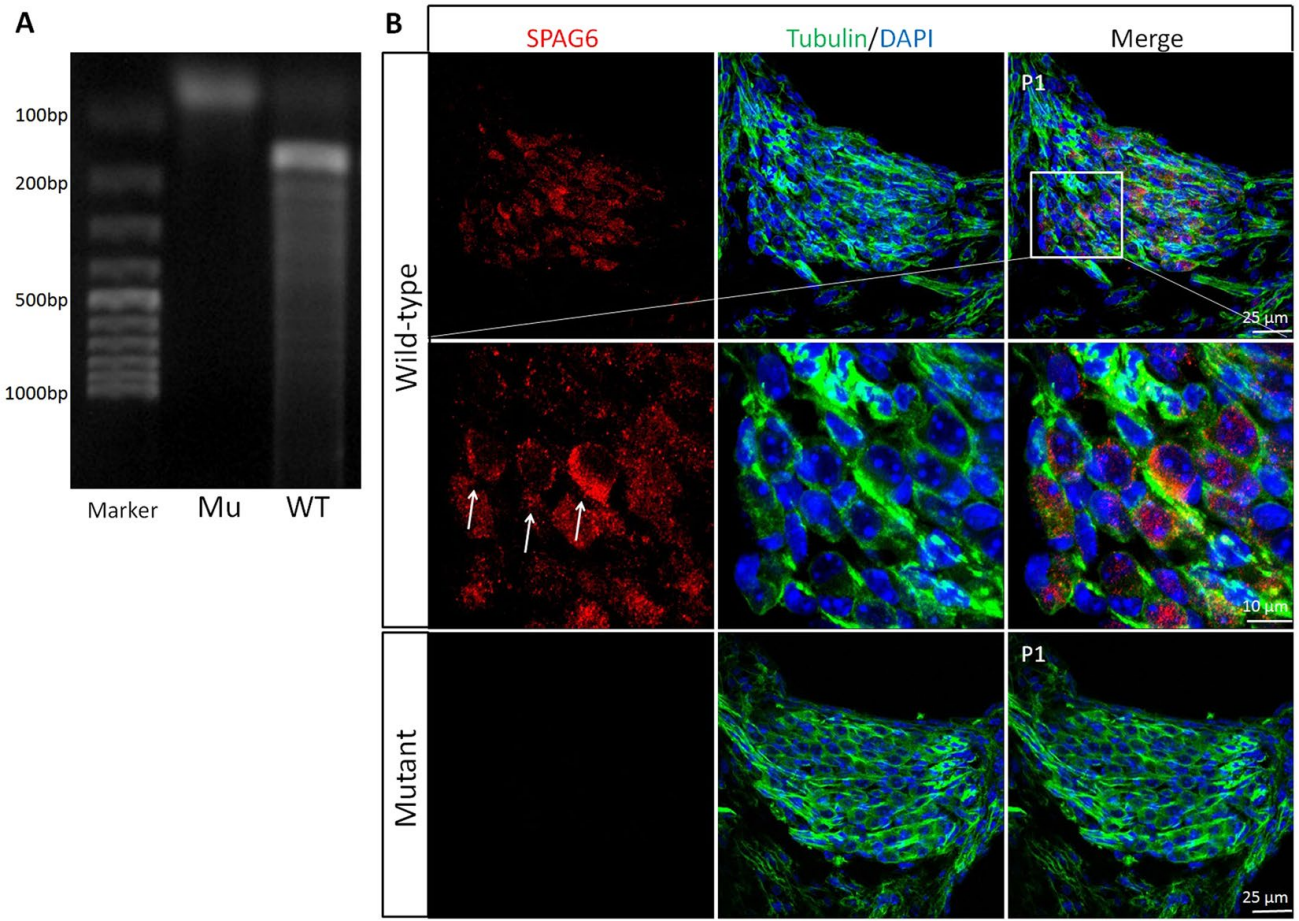
SPAG6 is expressed in mouse SGNs (**A**) RT-PCR showed SPAG6 expression in postnatal 1 day (P1) wild-type mouse SGNs at a band of 190 bp, while there was no detectable band in the mutants. (**B**) immunofluorescence showing positive staining of SPAG6 (red), β-tubulin Ш(green, neuronal specific marker) and DAPI (nucleus). SPAG6 was located primarily in the cytoplasm around the nucleus of SGNs (arrows) at P1.

The pattern of expression and role of SPAG6 in mouse SGNs was previously unknown. Therefore, the expression of SPAG6 was first examined. RT-PCR results showed SPAG6 expression in SGNs (Fig. 1A) and immunofluorescence result indicated that SPAG6 was located primarily in the cytoplasm around the nucleus of SGNs (Fig. 1B, arrows).

### Lower density of SGNs in Spag6 mutant mice

**Figure 2:**
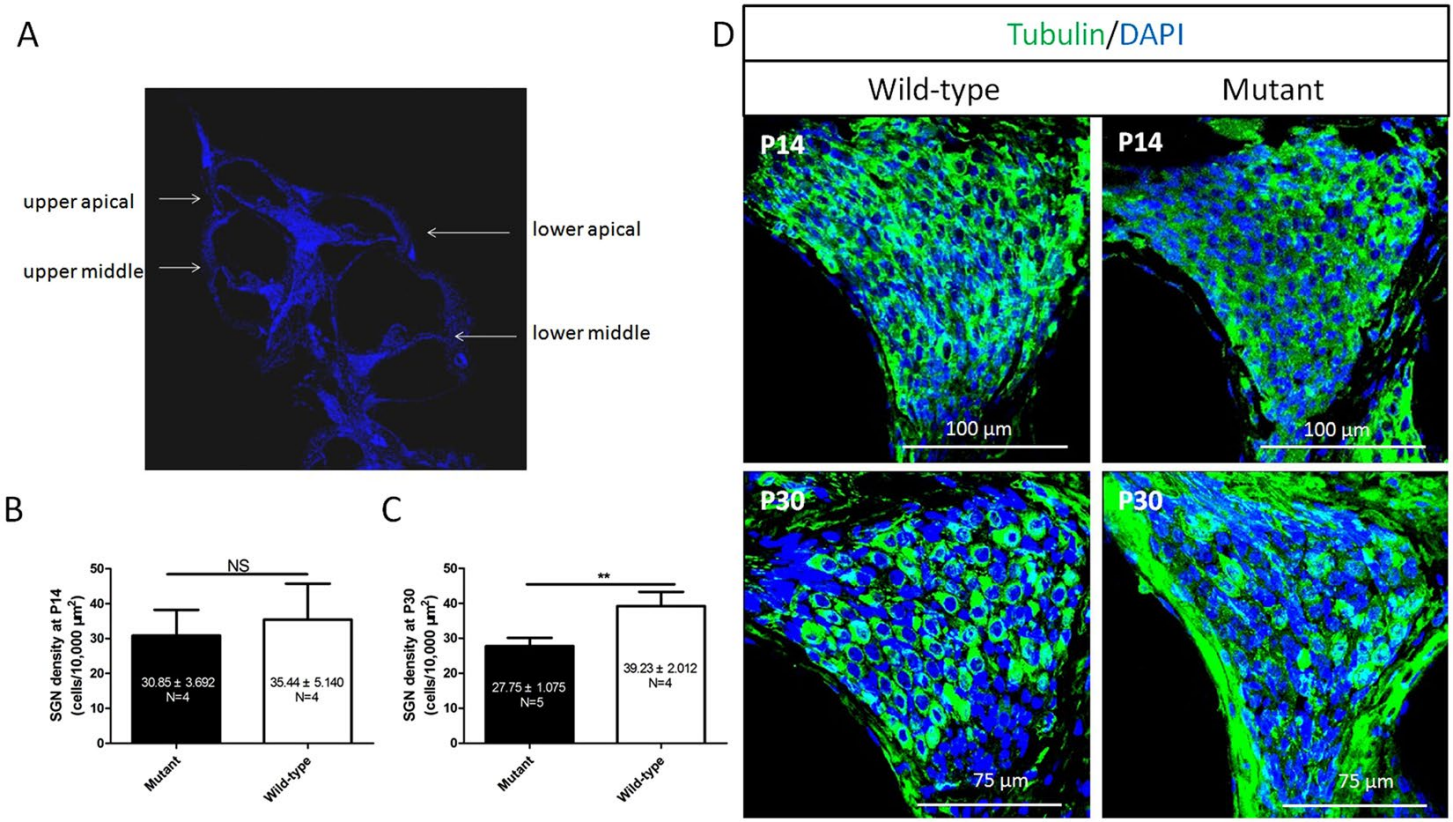
SGN density SGNs density at postnatal days 14 and 30 days (P14 and P30). (**A**) In order to reduce the error, only the upper middle turn in the frozen section through the modiolus was analyzed as illustrated. (**B**) At P14, there are no significant differences in SGN density between the mutant and wild-type mice (P > 0.05). (**C**) However, density was significantly different in SGN at P30 between the wild-type (39.23 ± 4.024, N = 4) and *Spag6* mutant mice (27.75 ± 2.403, N = 5)(P < 0.01). (**D**) Immunofluorescence staining of SGN with a neuron-specific marker (β-tubulin Ш) at P14 and P30. “N” indicates the number of mice. *P < 0.05, **P < 0.01, ***P < 0.001. Comparisons between groups were tested by Student’s t-test.

To elucidate the impact of SPAG6 deficiency on SGNs *in vivo*, SGNs density of the upper middle turn (Fig. 2A) was calculated at postnatal days 14 and 30 (P14 and P30), respectively. The data revealed no significant difference in SGN density between the mutant and wild-type mice at P14 (Fig. 2B). However, the mutants had a decreased density of SGNs at P30 compared to the wild-type mice (Fig. 2C), which was significant tested by Student’s t-test (P < 0.01).

### Increased apoptosis in SGNs of *Spag6* mutant mice

**Figure 3:**
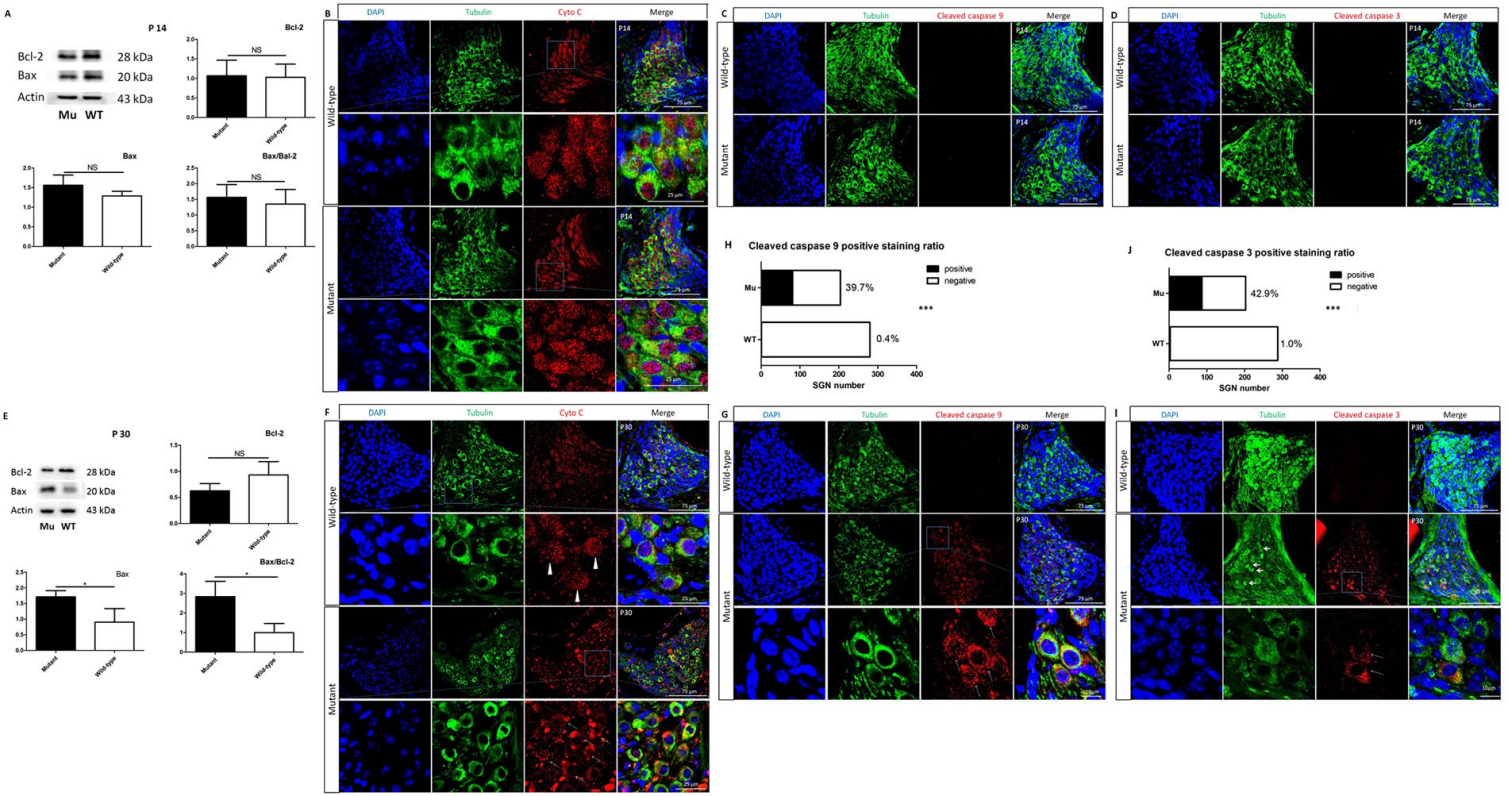
*Spag6* mutant SGNs apoptosis *in vivo* (**A**) Western blotting showed that at P14, there was no statistically significant difference in Bax or Bcl-2 expression, as well as and the ratio of Bax/Bcl-2. (**B**) Both wild-type and mutant mouse SGNs showed normal cytochrome c (red) distribution at P14. (**C**) No positive staining of cleaved caspase 9 could be found in control or mutant SGNs at day P14. (**D**) No positive staining of cleaved caspase 3 could be found in day P14 control or mutant SGNs. (**E**) Western blotting showed that at day P30, expression of Bax (mutant, 1.713 ± 0.1950, N = 3; wild-type, 0.9033 ± 0.4356, N = 3, P < 0.05) and Bax/Bcl-2 (mutant, 2.833 ± 0.7809, N = 3; wild-type, 0.9937 ± 0.4696, N = 3, P < 0.05) increased significantly in the mutant mice compared to the control group, while there was no significant difference in Bcl-2 expression between the two groups (P > 0.05). “N” indicates the number of mice. (**F**) In P30 wild-type mice SGNs, cytochrome c (red) was distributed uniformly (triangles). However, the distribution of cytochrome c (red) in the mutant SGN on day P30 was not uniform. The staining was assembled into aggregates (arrows). (**G**) The immunofluorescence showed positive staining of cleaved caspase-9 (red) in P30 *Spag6* mutant SGN, whereas in littermates few positive staining cells could be seen. (**H**) The positive staining ratio of cleaved caspase-9 in mutant SGNs was significantly increased (muant SGN, n = 204; wild-type SGN, N = 280). (**I**) Positive staining of cleaved caspase-3 (red, arrows) in P30 *Spag6* mutant mice was observed, whereas in littermates few positive staining cells were detected. In addition, abnormal SGN could be observed (green, arrows). (**J**) The positive staining ratio of cleaved caspase-3 was significantly higher than that of the control group (mutant SGN, N = 203; wild-type SGN, N = 288). *P < 0.05, **P < 0.01, ***P < 0.001. Comparisons between two groups in western blotting were tested by Student’s t-test. The rate comparison of cleaved caspase-9 and -3 positive staining between groups was analyzed by the chi-square test.

To determine why the SGN density was decreased in *Spag6*-deficient mice, western blotting and immunofluorescence staining were performed. The data showed increased Bax expression and an increased Bax/Bcl-2 (Fig. 3E, mutant, 2.833 ± 0.7809, N = 3; wild-type, 0.9937 ± 0.4696, N = 3), and an altered distribution of cytochrome c (Fig. 3F, arrows) in the mutant SGNs compared with their wild-type littermates at P30. Semi-quantitative analysis showed positive staining of cleaved caspase-9 and cleaved caspase-3 in *Spag6* mutant mice, whereas little positive staining could be found in the wild-type littermates at P30. The difference was significant (Fig. 3G). These results indicated that caspase–dependent apoptosis of SGNs had occurred at P30 in the mutant SGNs. However, There was no statistically significant difference in Bax, Blc-2, cleaved caspase-9 and cleaved caspase-3 levels, or cytochrome c distribution at P14 comparing mutant and wild-type groups (Fig. 3A–D), indicating minimal apoptosis.

**Figure 4:**
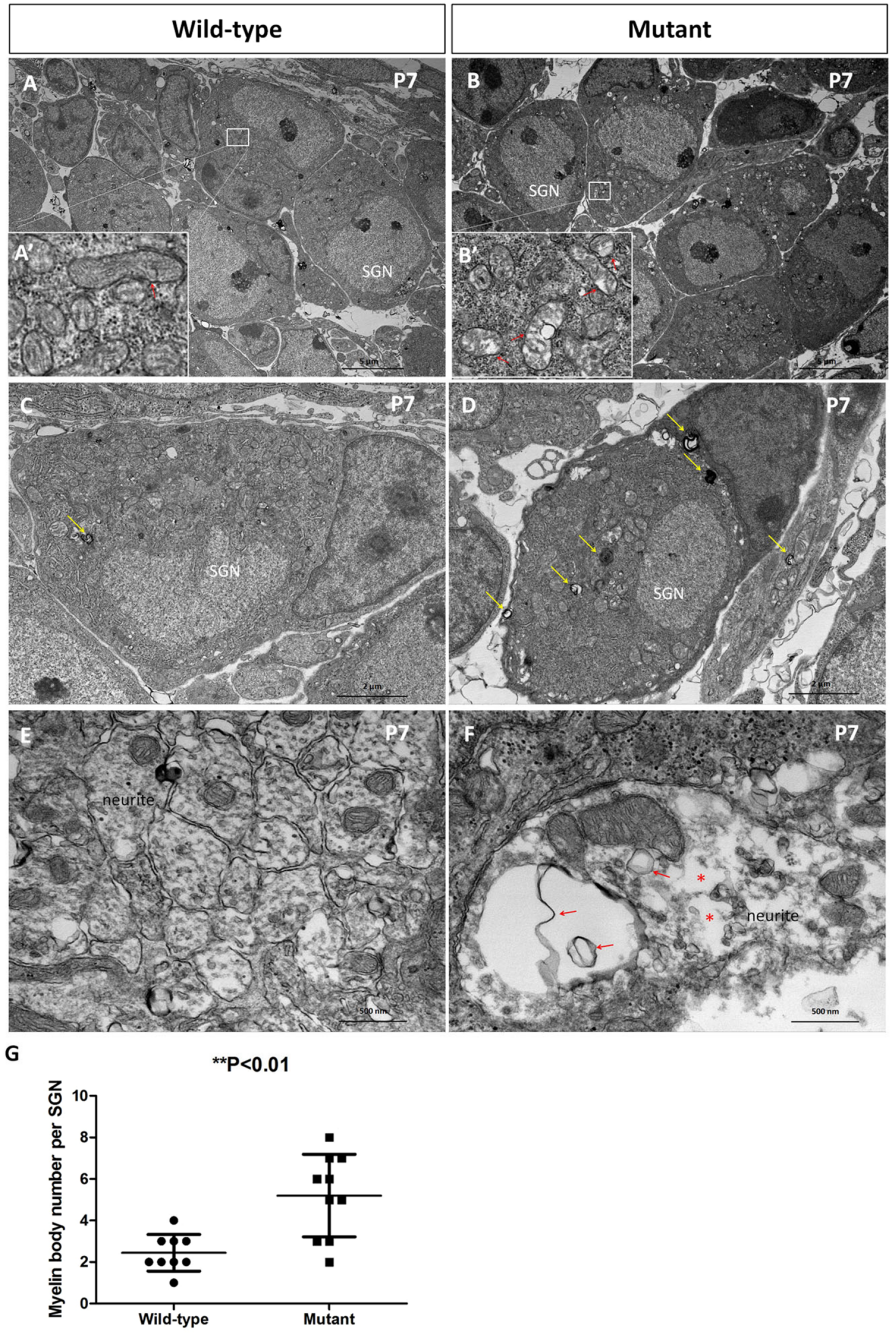
*Spag6* mutant SGNs showed defects in SGN ultrastructures at P7 Transmission electron microscopy (TEM) ultrastructure of P7 SGNs. (**A,B**) are lower magnification pictures of wild-type (**A**) and mutant (**B**) SGNs. A’ and B’ are magnified zones in (**A,B**) panels, respectively. Compared with the wild-type cells (A’), the cristae of mitochondria were sparse (red arrows in B’). There were many more myelin bodies in the mutant SGN soma (yellow arrows in **D**), whereas myelin bodies were only occasionlly seen in the wild-type SGN soma (yellow arrow in **C**). Furthermore, the mutant SGN neurites had relatively sparse microtubules and actin filaments (asterisks in **F**), as well as abnormal membranous structures (red arrows in **F**). The wild-type cells had a dense arrangement of microtubules and actin filaments in cross sections (**E**). No abnormal membranous structures were observed in the wild-type neurites. (**G**) The graph showed the difference between the wild-type and mutant in myelin body number, which was statistically significantly (wild-type, 2.444 ± 0.8819, n = 9; muant, 5.200 ± 1.9890, n = 10; **P < 0.01). “n” indicates the SGN number.

Although the mutant SGN at P14 seemed to be normal by immunostaining, transmission electron microscopy (TEM) revealed that at P7, the wild-type mitochondria were rich in cristae (Fig. 4A’, red arrow), while the cristae of mutant mitochondria were sparse (Fig. 4B’, red arrows). there were more myelin bodies in the mutant SGN soma (Fig. 4D, yellow arrows), indicating membranous abnormalities of some organelles, whereas myelin bodies were only occasionlly seen in the wild-type SGN soma, we analyzed the myelin body number in each SGN and the difference was statistically significant (wild-type, 2.444 ± 0.8819, n = 9; muant, 5.200 ± 1.9890, n = 10; **P < 0.01). Furthermore, the mutant SGN neurites had relatively sparse microtubules and actin filaments, as well as other abnormal membranous structures (Fig. 4F, red arrows), which was consistent with the differences in myelin bodies in the soma. The wild-type SGNs had a dense arrangement of microtubules and actin filaments in cross sections. Few abnormal membranous ultrastructures were observed in the wild-type neurites. All of these observations suggest that abnormalities in SGNs caused by *Spag6* knockout appear by P7, before SGN apoptosis.

### Lack of SPAG6 affects the growth of SGNs in explants

**Figure 5:**
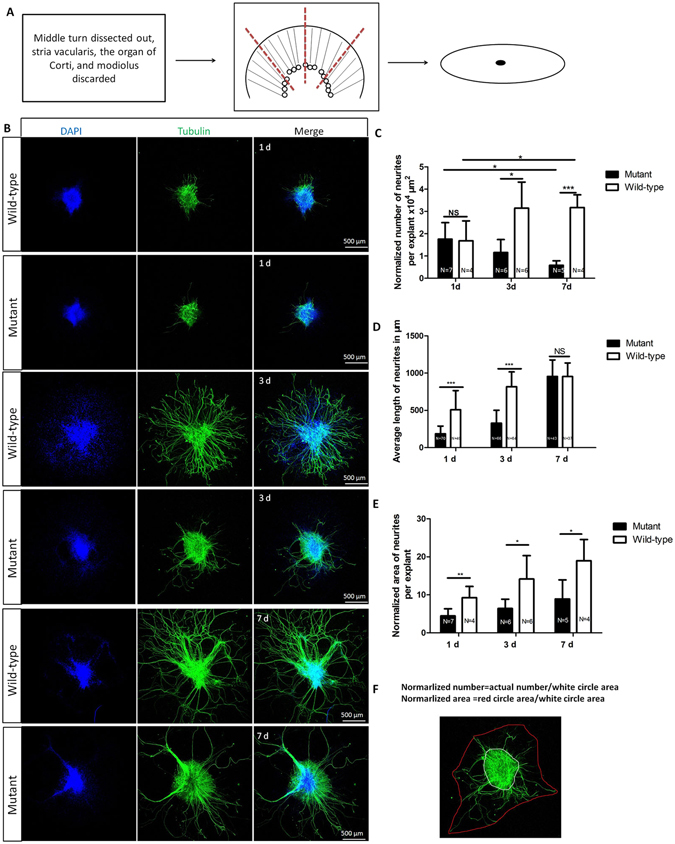
Lack of SPAG6 affects the growth of SGNs *in vitro* The schematic of dissection and culture of SGN explants form neonatal mouse cochleae. The middle turn of P3 cochlea was dissected out, the strial vacularis, the organ of Corti, and the modiolus were discarded, and remnant SGN bulk was cut into four equal pieces, attached to coverslips, and cultured. (**B**) Neurite regrowth from *Spag6* wild-type and mutant mice in SGNs explants in 3D culture for 1–7 days. (**C**) The normalized number of the regenerating neurites were compared between the mutant and wild-type mouse SGN explants. At1 d, the mutant SGN explants showed no obvious difference in normalized regenerating neurite number compared with wild-type SGNs (mutant, 1.7560 ± 0.7404, N = 7; wild-type, 1.6855 ± 0.8923, N = 4, P > 0.05). Nevertheless, at 3 d (mutant, 1.1576 ± 0.5817, N = 6; wild-type, 3.1477 ± 1.1703, N = 6, P < 0.05) and 7 d (mutant, 0.5802 ± 0.2070, N = 5; wild-type, 3.1759 ± 0.5734, N = 4, P < 0.001) the mutant SGN neurite number decreased compared to the wild-type explants. Furthermore, in the wild-type SGNs explants, the normalized neurite number at 7 d was significantly higher than that at 1 d (P < 0.05). However, in the *Spag6* mutant SGN explants, the normalized neurite number at 7 d was significantly less than that at 1 d (P < 0.05). (**D**). The length of regrowth neurites was measured. At 1 d and 3 d, the SGN expants from mutant mice showed shorter neurites than the control group (P < 0.001, P < 0.001). However, at 7 d, there was no significant difference in neurite length. E. The normalized total surface occupied by regenerating neurites was analyzed. The mean area at 1 d (mutant, 4.4587 ± 1.8765, n = 7;wild-type, 9.2646 ± 2.9597, n = 4, P < 0.01), 3 d (mutant, 6.4202 ± 2.4323, n = 6;wild-type, 14.1673 ± 6.1332, n = 6, P < 0.05)and 7 d (mutant, 8.9067 ± 5.0314, n = 5; wild-type, 18.9988 ± 5.5690, n = 4, P < 0.05) were different between mutant and wild-type SGNs. “n” indicates the SGN explant number. F. The white irregular shape means SGN explant area. The red irregular shape means the actual area occupied by neurites. The normalized number of neurites in C is a ratio of actual neurite number to white irregular shape area. The normalized area of neurites in D is a ratio of red irregular shape area to the white irregular shape area. *P < 0.05, **P < 0.01, ***P < 0.001. Comparisons between mutant and wild-type groups in the same time point were tested by Student’s t-test (Fig. 5C, D and E). Pairwise comparison between three time points in the same group were analyzed using a one-way ANOVA followed by Newman–Keuls post-hoc analyses (Fig. 5C).

To explore the role of SPAG6 in SGNs before apoptosis, SGN explants were prepared from P3 wild-type and mutant mice and kept in 3D culture for 1–7 days (d) (Fig. 4A and B). We then analyzed three parameters that allowed for a more precise comparison between neurite regrowth from mutant versus control SGNs. First, we measured the normalized number of regenerating neurites (Fig. 4C). As illustrated in Fig. 5F, the normalized number is the ratio of the actual number to the SGN bulk area. At 1 d, the mutant SGN explants showed no obvious difference in regenerating neurite number between the two groups. Nevertheless, at 3 d and 7 d, the mutant SGN neurite normalized number decreased significantly (P < 0.05 and P < 0.001, respectively) compared to the wild-type explants, which suggested a decrease in regrowth neurite density in SGN bulks. Furthermore, in the wild-type SGNs explants, the normalized neurite number at 7 d is significantly higher than that at 1 d (P < 0.05). However, in the *Spag6* mutant SGN explants, the normalized neurite number at 7 d is significantly less than that at 1 d (P < 0.05). Under normal conditions, the SGN bulk was sticking onto the coverslides, cells of the SGN explant grew out from the explant gradually from day 1 to day 7, which expanded the explant area. At the same time, SGN neurites lengthened and so could be counted. In the mutant SGN, the actual number at day 7 was more than that on day 1, but because of the larger bulk area, the normalized neurite number at day 7 is less than day 1. This could be interpreted as a decrease in SGN neurite density in the explant bulk.

Second, we measured the length of regrowth neurites (Fig. 5D). At 1 d and 3 d, the SGNs explants from mutant mice showed shorter neurites than the wild-type group (P < 0.001, P < 0.001). This finding could be attributed to a rate of neurite growth that is lower for neurites lacking SPAG6. However, there was no significant difference in neurite length at 7 d. This could be explained by Spag6-deficiency only slowing the growth rate, but not changing the final length of neurites at day 7. Another possible explanation is that with elongation wild-type neurites cross over eachother and have many branches. This lateral cross growth could have reduced the length of wild-type neurites.

Third, the normalized area occupied by regenerating neurites was analyzed (Fig. 5E). The normalized area means the ratio of red circle area to the white circle area (Fig. 5F). The mean normalized areas at different time points all turned out to be different between mutant and wild-type SGNs (P < 0.01, P < 0.05, P < 0.05).

### SPAG6 deficiency impairs the growth of growth cones

**Figure 6:**
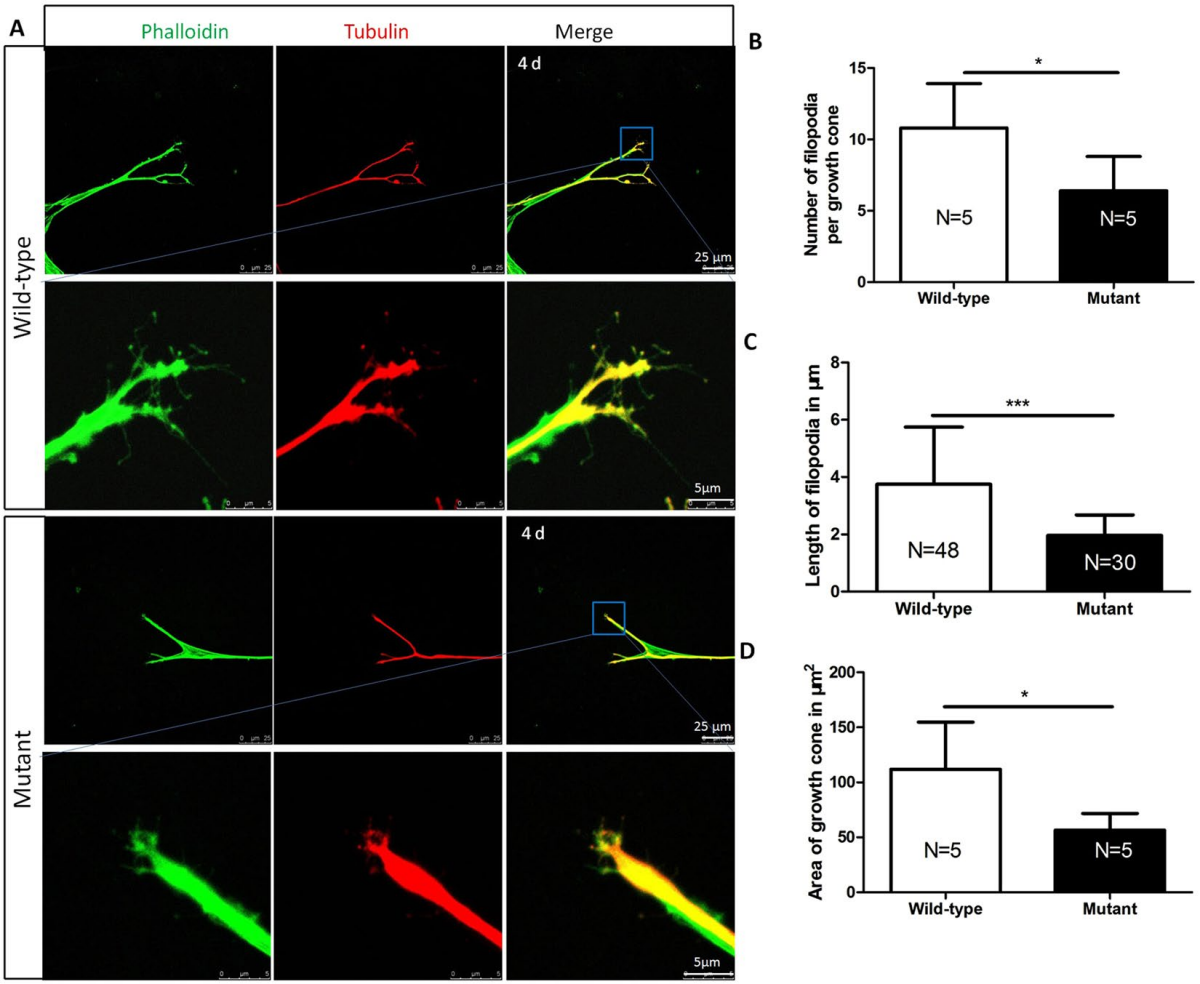
SPAG6 deficiency impairs the growth of growth cones (**A**) Morphology of the SGNs growth cone of *Spag6* mutant and wild-type mice. Phalloidin, green; β-tubulin, red. (**B**) Quantification of the average number of filopodia per growth cone (wild-type, 10.80 ± 3.114, N = 5; 6.400 ± 2.408 N = 5; *P < 0.05). (**C**) Quantification of the average length of filopodia from the edge of the growth cone to tips of each filopodia (wild-type, 3.741 ± 2.000, N = 48; 1.964 ± 0.720, N = 30; “N” indicates the number of growth cones; ***P < 0.001). D. Quantification of the average area per growth cone (wild-type, 111.9 ± 42.88, N = 5; mutant, 56.63 ± 15.19, N = 5; *P < 0.05). Comparisons between two groups were tested by Student’s t-test.

The growth cone is a dynamic extension of a developing neurite seeking its synaptic target. Its peripheral domain and central domain are primarily composed of actin and microtubules, respectively. SPAG6 disturbed both microtubules and actin in our previous studies^17^, and TEM showed a sparse arrangement of microtubules and actin in cross section of SGN neurites. Therefore, the effects of SPAG6 on the SGN growth cones were investigated by comparing the number, length of filopodia, and the area of growth cone after culture for 4 d (Fig. 6A). We stained the cultured SGN with phalloidin and β-tubulin Ш and found that the average number of filopodia emerging from the SPAG6 mutant growth cones was less compared to the wild-type group (Fig. 6B, P < 0.05). Similarly, the average filopodia length from the tips of individual filopodia to the edge of the growth cone was shorter (Fig. 6C, P < 0.001), and the growth cone area of SPAG6 mutant neurites was smaller than those of wild-type ones (Fig. 6D, P < 0.05). Altogether, SPAG6 deficiency impaired the growth of growth cones and the development of filopodia compared to the wild-type groups.

### SPAG6 deficiency decreases synapse density in SGN explants

**Figure 7:**
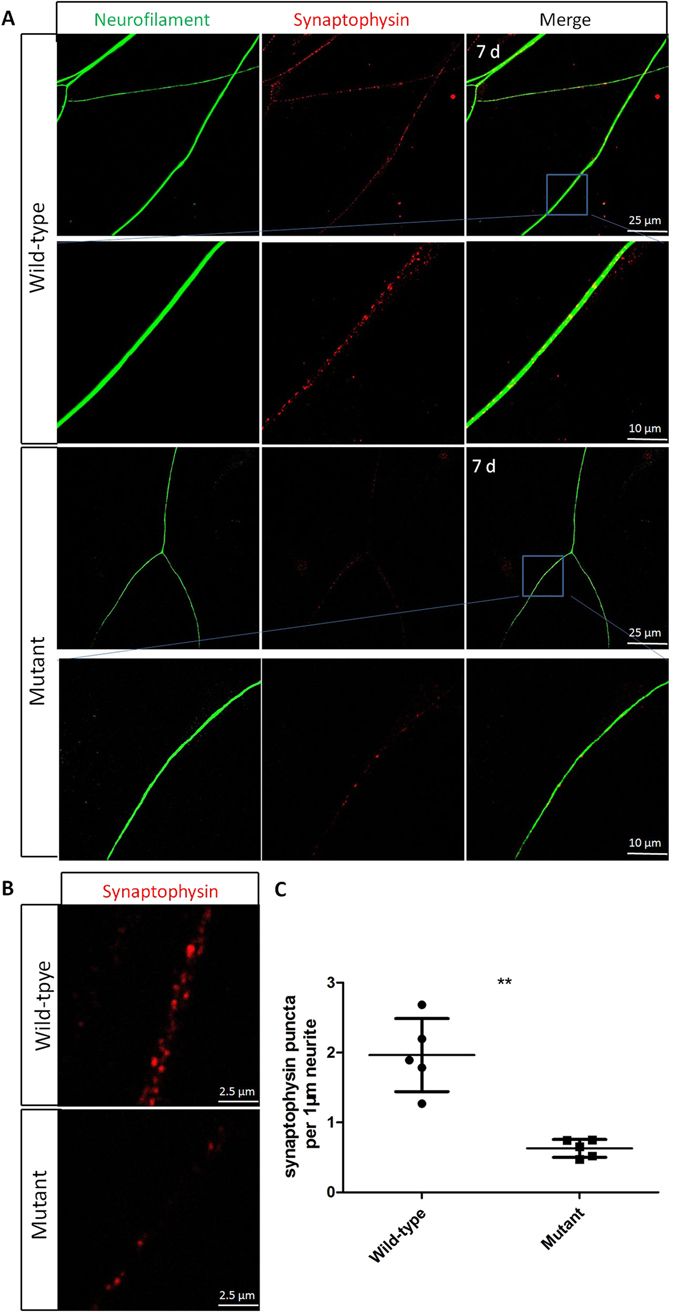
SPAG6 deficiency decreases synapse density in SGN explants (**A**) Representative images of SGNs explants from *Spag6* mutant mice and wild-type mice immunostained with neurofilament (green) and synaptophysin (red). (**B**) Representative images in high magnification of synaptophysin (red) immunostained SGN explants from *Spag6* mutant mice and wild-type mice. (**C**) Quantification of the number of synapse puncta in the mutant and wild-type SGN explants. (wild-type,1.964 ± 0.5223, N = 5; mutant, 0.6279 ± 0.1279, N = 5; “N” indicates the number of individual experiments **P < 0.01). Comparisons between two groups were tested by Student’s t-test.

To further investigate the effect of SPAG6 on the function of organotypic SGN cultures, we detected synaptophysin expression in neurites to analyze the synapse density. Synaptophysin is a synaptic vesicle glycoprotein with four transmembrane domains and correlates directly with the presence of neurotransmitter^16, 17^. Therefore, it also serves as a specific presynapse marker. Representative images after culture for 7 days are shown in Fig. 7. Statistical analysis demonstrated that synaptophysin punctate density was significantly decreased in the mutant compared to wild-type mice (P < 0.01).

### Higher sensitivity of the *Spag6* mutant SGNs to paclitaxel

**Figure 8:**
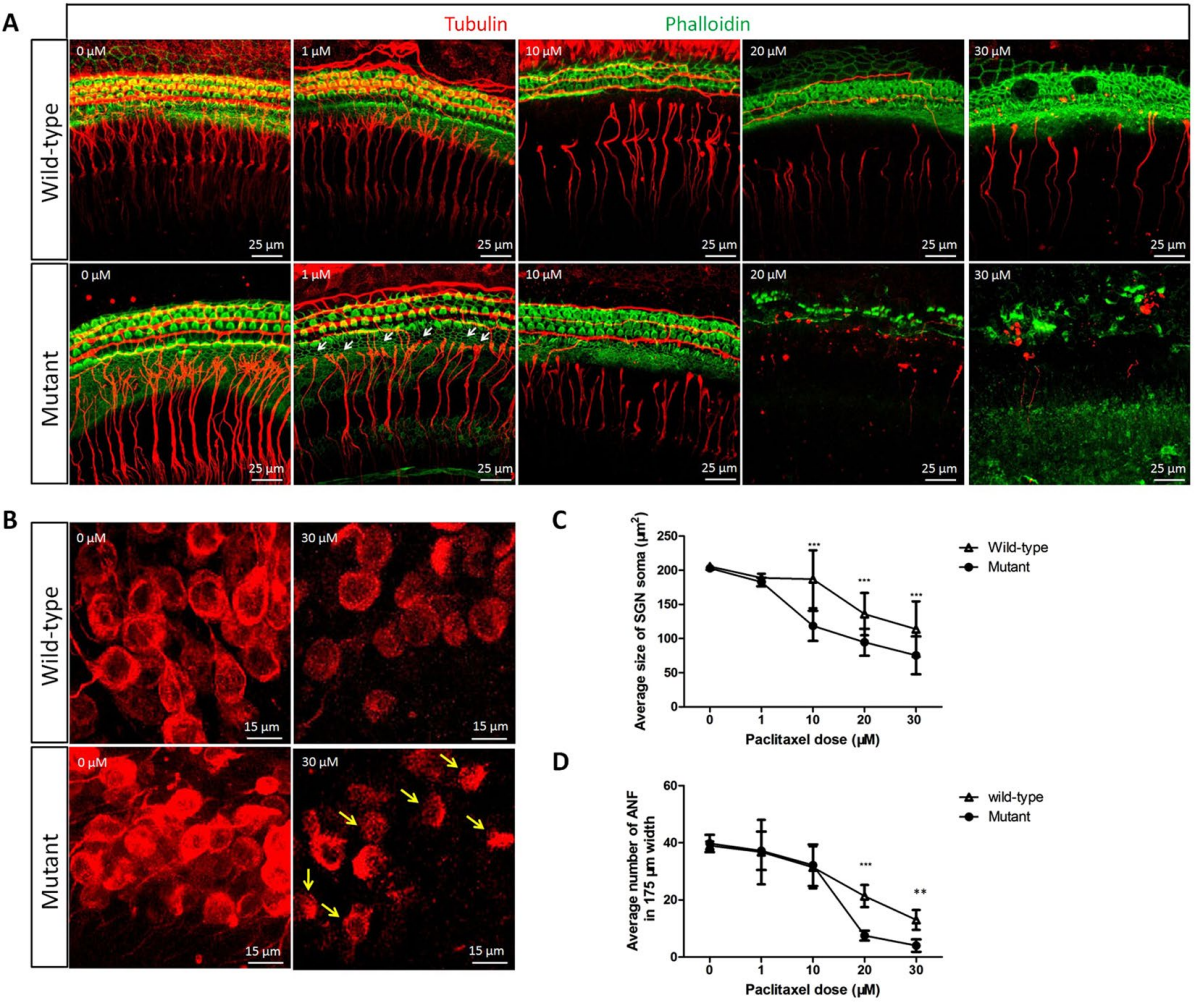
Higher sensitivity of *Spag6* mutant SGNs to paclitaxel (**A**) Photomicrographs show SGN organotypic cultures from the upper basal turn of the cochlea after 48 hours of treatment with paclitaxel; concentration is shown in each panel. The mutant and wild-type auditory nerve fibers (ANFs) extending radially toward the hair cells at 0 μM. Note that, although there was no statistic difference in ANF number and soma size at 1 μM, swelling terminals of ANFs can be seen in the mutant SGN (white arrows) while it was not obvious in the wild-type group; at 10 μM, although the number of ANF is the same, the length of mutant ANF has changed a lot and become much shorter, the terminals of the ANF were far from hair cells according to the observation. And at the same time the soma size decreased, the size of SGN soma were much smaller in the mutant mice (C, wild-type, 186.8812 ± 42.44, n = 86; mutant, 118.7140 ± 22.06, n = 60; P < 0.001). Loss of ANFs as dose increases from 20 μM to 30 μM, the mutant conserved less ANF number (20 μM: wild-type, 21.3333 ± 3.88, n = 6; mutant, 7.5000 ± 1.73, n = 4; P < 0.001; 30 μM: wild-type, 13.0000 ± 3.46, n = 6; mutant, 4.0000 ± 2.16, n = 4; “n” indicates SGN explant number; P < 0.01) and smaller soma size wild-type (20 μM: wild-type, 135.8365 ± 30.92, n = 41; mutant, 94.5951 ± 19.73, n = 82; P < 0.001; 30 μM: wild-type, 113.8856 ± 40.64, n = 31; mutant, 75.5018 ± 27.78, n = 99; “n” indicates SGN soma number; P < 0.001) than those of the wild-types, and the differences were significant (**D**). (**B**) Representative photomicrographs of normal SGN with large, round, densely package SGN. At 30 μM paclitaxel the mutant showed more condensed SGNs (yellow arrows) and decreased number of SGN than those of the wild-type mice. *P < 0.05, **P < 0.01, ***P < 0.001. Comparisons between two groups in the same drug dose were tested by Student’s t-test.

In our previous study, *Spag6*-deficient mouse embryonic fibroblasts (MEFs) were more sensitive to paclitaxel and showed an increased cell death rate^17^, which led us to investigate the impact of SPAG6 on SNGs response to different concentrations of paclitaxel, a microtubule stabilizing agent that is ototoxic to rat cochear organotypic cultures^20^. As shown in Fig. 8, the number of surviving nerve fiber bundles and SGN soma size were quantified with different doses of paclitaxel exposure. Approximately 40 fibers/175 mm are present both in untreated mutant and wild-type cultures (0 μM). As the dose of paclitaxel increased, the damage was more severe. At 1 μM, although there was not a statistically significant difference in auditory nerve fiber (ANF) number and soma size, swelling in the terminals of ANFs could be seen in the mutant SGN (white arrows), while it was not obvious in the wild-type group. At 10 μM, although the numbers of ANF were similar, the length of mutant ANF had become much shorter, and the terminals of the ANF were far from hair cells. At the same time, the soma size decreased. The 48 hour of paclitaxel treatment caused a statistically significant decline in the number of *Spag6* mutant nerve fibers and the size of SGN soma at 20 and 30 μM compared to the wild-type group (Fig. 8).

## Discussion

Mice do not have an intact auditory pathway and mature auditory function until P21 to P30. We wanted to determine the impact of SPAG6 deficiency on mature SGN. Therefore, SGN were studied at P30. Our data showed that Spag6 mutant SGN density is lower than that of the wild-type mice at P30, probably as a result, at least in part, of increased apoptosis. Although the mechanism underlying apoptosis in *Spag6* mutant SGNs needs further investigation, increased expression of Bax and the ratio of Bax and Bcl-2, the uneven distribution of cytochrome c and activated caspase 9 and 3 positive staining suggests that apoptosis was mediated through the mitochondrial pathway. Interestingly, Bcl-2 was not changed or increased significantly between the two groups, although Bax and Bcl-2 often show opposite trends in apoptosis related models^22, 23^. Therefore, it seems that *Spag6* increases Bax to alter the balance between Bax and Bcl-2, rather than decreasing Bcl-2. Bax could bind to mitochondrial membranes, cause mitochondrial membrane permeability changes and release of cytochrome c, activating pro-apoptosis factors like caspase 9 and caspase 3, finally leading to apoptosis. It has been reported that knockdown of SPAG6 increases the expression of caspase −3, −9 and −8 in human SKM-1 and K562, which is in accord with our previous results^9^.

A recent study reported that SPAG6 may regulate apoptosis in SKM-1 cells through the tumor necrosis factor-related apoptosis-inducing ligand (TRAIL) signal pathway^24^. In this paper, inhibition or overpression of SPAG6 could activiate or inhibit, respectively, the TRAIL signal pathway, indicating a possible role for SPAG6 in the extrinsic apoptotic pathway. TRAIL is a proapoptotic ligand which can activate proapoptotic receptors on the cell surface and initiate the extrinsic apoptotic pathway^25^. Crosstalk between the extrinsic and intrinsic pathways occurs through active caspase 8, which can cleave Bid to tBid. tBid translocates to the mitochondria and interacts with Bax/Bak, leading to mitochondrial outer membrane permeabilization^25^. Due to crosstalk between pathways, it is not yet possible to definitively assign the mechanism of apoptosis in SGN to either the extrinsic or intrinsic pathway. Another possibility is that SPAG6 leads to apoptosis through both pathways.

We showed apoptosis at P30. The next question was when does apoptosis begin? We chose a mid-time point (from birth to P30), P14, and found that at P14, apoptosis had not yet occurred. Although there was no obvious decrease in the mutant SGN density at P14, TEM showed that *Spag6* mutation brought about obvious pathologic changes in SGN ultrastructure at P7, such as increased myelin bodies in the cytoplasm, less mitochondrial cristae, and sparse arrangements of microtubules and actin. Therefore, it is suggested that *Spag6* mutation could affect a number of ultrastructural features of SGNs as early as P7. These abnormalities might happen at an even earlier time. It is worth noting that mitochondria, the most prominent roles of which are to produce the energy for the cell in the form of ATP, and to regulate cellular metabolism. Numerous cristae expand the surface area of the inner mitochondrial membrane, enhancing the ability to produce ATP. It is reported that availability of ATP changes in a cell cycle-specific manner. The relationship between cellular proliferation and mitochondria has been investigated using HeLa cells. Tumor cells require an ample amount of ATP in order to synthesize bioactive compounds such as lipids, proteins, and nucleotides for rapid cell proliferation^26^. The variation in ATP levels at different stages of the cell cycle support the hypothesis that mitochondria play an important role in cell cycle regulation^27^. The role of *Spag6* in cell proliferation has been reported^28^. It is possible that *Spag6* may regulate proliferation through effects on mitochondria.

A 3D culture system, which can encapsulate SGNs and mimic the normal 3D environment *in vivo* is regarded as the ideal model to preserve the delicate structure and function of SGN explants. Our team members previously succeeded in establishing the 3D culture system with matrigel to encapsulate the spiral ganglion region isolated from neonatal mice^29^. Because mouse cochlea gradually ossify from birth to P7, the time point for SGN 3D culture was selected at P3–P5. We analyzed the development of both wild-type and mutant SGNs explant *ex vivo*. The data suggested impaired growth, including the growth of neurites and growth cones, and decreased synapse density in the mutant SGN explants compared with the wild-type groups, indicating that SPAG6 mutation disturbs SGN development and function.

Neuronal growth cones are situated on the very tips of axons and dendrites. The peripheral domain is composed primarily of an actin-based cytoskeleton, and contains the filopodia which are highly dynamic. The central domain is located in the center of the growth cone and is composed primarily of a microtubule-based cytoskeleton, and contains many organelles and vesicles^30, 31^. During growth cone turning, microtubules extend from the central domain preferentially into one side of the peripheral domain, which needed accurate cross talk between microtubule and actin. Therefore, the abnormality of microtubule and actin regulating protein could both lead to abnormal growth cones. Study found that neurons lacking of microtubule-associated protein 1B showed defect in both growth cone turning and axonal branch formation^32, 33^. Silencing of CRMP-5, a protein interacting with tubulin, led to abnormal growth cone morphology in neurons^34, 35^. In this study, disrupted microtubules and actin filaments were observed, which possibly resulted in defects in the growth cone. Our previous study also found an abnormal distribution of microtubules and actin in the *Spag6*-deficient (KO) MEFs during migration^17^.

Microtubules are also known to form the transport pathways for cargo trafficking in axons and dendrites in mature neurons^36–38^. Synaptophysin, a glycoprotein present in the membrane of presynaptic vesicles^39^, was used as the presynapse marker in our research. In neurons, synaptophysin are directed via microtubules by coupling to dynein motor. It is believed to modulate the efficiency of the synaptic vesicle cycle and affect higher brain functions^40^. Our data revealed that *Spag6* knockout decreased the density of synaptophysin *in vitro*, demonstrating that mutant SGN function was impaired to some extent, which is thought to be related to disabled microtubule-based vesicle transport in the *Spag6* mutant SNG explants. This observation is consistent with the previous results that much vesicles were observed in KO MEFs^17^.

Paclitaxel, a microtubule stabilizer, is an antineoplastic drug widely used for the treatment of malignant tumors. Several reports suggest that paclitaxel can cause mild to moderate sensorineural hearing loss^41^. Paclitaxel damages cochlear hair cells in a dose dependent manner and also damaged auditory nerve fibers (ANF) and SGN in rats^20^. Because there is no report of ototoxity of paclitaxel in mouse cochlear organotypic cultures, the paclitaxel dose used in rat cochlear organotypic cultures was used for reference^20^. The results of this study showed a similar trend with that in rat cochlear organotypic cultures. ANF and SGN soma in the two groups were evaluated, and the results are consistent with the response in wild-type and mutant MEFs^17^. Mutant SGN showed a higher sensitivity to paclitaxel than the wild-type cells.

In conclusion, the present study illustrates some functions of *Spag6* in SGN. Our findings demonstrated that *Spag6* deficiency leads to abnormal SGN ultrastructure at day P7 and apoptosis of SGN at P30. Furthermore, we showed that the lack of *Spag6* impairs SGN development and function *in vitro*. These findings provide novel insights into the role of *Spag6* in the inner ear SGN.
